# 

**Published:** 1889-03

**Authors:** 


### THE BRISTOL

### Medico-Chirurgical Journal.

A JOURNAL OF THE MEDICAL SCIENCES FOR THE WEST OF ENGLAND AND SOUTH WALES

*PUBLISHED UNDER THE AUSPICES OF*

*THE BRISTOL MEDICO-CHIRURGICAL SOCIETY.*

EDITOR:
J. GREIG SMITH, M.A., F.R.S.E.

ASSISTANT-EDITOR:
L. M. GRIFFITHS.

---

"Scire est nescire, nisi id me
Scire alius sciret."

---

*VOL. VII.*

BRISTOL: J. W. ARROWSMITH;

LONDON: J. & A. CHURCHILL, 11 NEW BURLINGTON STREET.

1889.

L.H.

R31
.B8

### CONTENTS OF VOLUME VII.

ORIGINAL PAPERS: *Page*

Hydrate of Chloral: A Therapeutic Study. By JOHN KENT SPENDER, M.D. ... ... ... ... ... ... ... ... ... ... 1

Dilatation of the Stomach. By J. MICHELL CLARKE, M.A., M.B. ... ... ... ... ... ... ... ... ... ... ... ... 21

Observations on the Normal Diet. By G. MUNRO SMITH ... ... 73

On the Treatment of Laryngeal Growths. By BARCLAY J. BARON, M.B. ... ... ... ... ... ... ... ... ... ... ... 78

Reminiscences of an Army Surgeon. By WILLIAM JOHNSTONE FYFFE, M.D. ... ... ... ... ... ... ... .. ... ... ... 145

Accidental Hæmorrhage. By J. G. SWAYNE, M.D. ... ... ... 170

Some Surgical Aspects of Carcinoma. By NELSON C. DOBSON 225

Inebriety. By ALFRED J. H. CRESPI ... ... ... ... ... ... 244

CLINICAL RECORDS:

On the Treatment of Acute Inflammations of the Middle Ear. By CHARLES F. PICKERING ... ... ... ... ... ... ... 32

On "Birth Palsy," with Illustrative Cases. By F. W. JOLLYE 87

Cases of Oral Surgery. By NELSON C. DOBSON ... ... ... ... ... 108

Chronic Pharyngitis with Elongated Uvula. By P. WATSON WILLIAMS M.B. ... ... ... ... ... ... ... ... ... ... 189

Page

Tubercle of Medulla Oblongata. By J. Michell Clarke, M.A., M.B. ... 194

A Case of Myxœdema. Congenital Heart Disease. Disseminated Sclerosis. By J. Michell Clarke, M.A., M.B. 258

Health Resorts:

Bournemouth. By Alfred J. H. Crespi ... 36

Clifton. By John Beddoe, M.D., F.R.S. ... 114

Weston-super-Mare. By Charles Vernon Hitchins ... 198

Visit of the British Medical Association to Bristol in 1891 ... 142

The International Medical Congress ... 214

Reviews of Books ... 44, 123, 207, 267

Notes on Preparations for the Sick ... 56, 131, 215, 276

Periscope ... 60, 134, 217, 280

Scraps ... 71, 143, 223, 287

### Bristol Medico-Chirurgical Society.

### President.

R. SHINGLETON SMITH, M.D., B.Sc. Lond.

### President-Elect.

NELSON C. DOBSON.

### Honorary Secretary.

G. MUNRO SMITH.

### Committee.

*Past Presidents.*
R. W. COE.
AUGUSTIN PRICHARD, M.D. Berlin.
J. G. SWAYNE, M.D. Lond.
E. LONG FOX, M.D. Oxon.
JAMES G. DAVEY, M.D. St. And.
W. JOHNSTONE FYFFE, M.D. Dub.
G. F. BURDER, M.D. Aberd.
W. H. SPENCER, M.D., M.A. Cantab.
F. POOLE LANSDOWN.
L. M. GRIFFITHS.

F. RICHARDSON CROSS, M.B. Lond.
A. J. HARRISON, M.B. Lond.
W. H. HARSANT.
E. MARKHAM SKERRITT, M.D., B.S., B.A. Lond.
J. E. SHAW, M.B., C.M. Ed.
J. GREIG SMITH, M.B., C.M., M.A. Aberd., F.R.S.E.

Medical Men wishing to join the Society are requested to communicate with the Honorary Secretary, G. MUNRO SMITH, 70 Pembroke Road, Clifton, Bristol. The Annual Subscription is 10/6.

---

*Extracts from Journal By-laws.*

4.—The *Journal* shall be delivered free to Subscribers and also to Members of the Society whose subscriptions are not in arrear.

6.—The Annual Subscription to the *Journal* for those who pay before the 1st of July shall be 5/-, post free, or 6/- if paid after that date.

---

Communications intended for insertion in the *Journal* should be sent to the Editor, J. GREIG SMITH, M.A., F.R.S.E., 16 Victoria Square, Clifton, Bristol.

Books for Review, Exchange Journals, and communications referring to the delivery of the *Journal* should be sent to the Assistant-Editor, L. M. GRIFFITHS, 9 Gordon Road, Clifton, Bristol, to whom Subscriptions are to be paid *in advance*.

Authors requiring Reprints of their Papers should communicate with the Publisher.

Cases for Binding Vols. I., II., III., IV., V., and VI. are now ready, and may be obtained, price 7*d.* each (postage 2*d.* extra), upon application to J. W. ARROWSMITH, Quay Street, Bristol; or the *Journal* will be bound, including Case, for 1/3 per volume.

### Bristol Medico-Chirurgical Society.

---

### PAST PRESIDENTS.

1874—75. FREDERICK BRITTAN, M.D., B.A. Dub.
1875—76. ROBERT WILLIAM COE.
1876—77. SAMUEL MARTYN, M.D. Ed.
1877—78. AUGUSTIN PRICHARD, M.D. Berlin.
1878—79. HENRY EDWARD FRIPP, M.D. Würzburg.
1879—80. WILLIAM MICHELL CLARKE.
1880—81. JOSEPH GRIFFITHS SWAYNE, M.D. Lond.
1881—82. EDWARD LONG FOX, M.D. Oxon.
1882—83. JAMES GEORGE DAVEY, M.D. St. And.
1883—84. WILLIAM JOHNSTONE FYFFE, M.D. Dub.
1884—85. GEORGE FORSTER BURDER, M.D. Aberd.
1885—86. WILLIAM HENRY SPENCER, M.D., M.A. Cantab.
1886—87. FRANCIS POOLE LANSDOWN.
1887—88. LEMUEL MATTHEWS GRIFFITHS.

1889.

### LIST OF SUBSCRIBERS

TO THE

### Bristol Medico-Chirurgical Journal.

*Those marked * are Members of the Society.*

| *Name.* | *Residence.* |
| --- | --- |
| Abercrombie, John, M.D. Cantab.... ... ... | 23 Upper Wimpole Street, London, W. |
| Ackland, J. Mackno ... ... ... ... ... ... | 24 Southernhay, Exeter. |
| Ackland, W. H., M.D. St. And. ... ... ... | Bideford. |
| *Ackland, W. R. ... ... ... ... ... ... ... | 5 Rodney Place, Clifton. |
| Adams, George ... ... ... ... ... ... ... | 11 Westbourne Place, Clifton. |
| *Adams, John Barfield ... ... ... ... ... ... | 10 Cotham Brow, Bristol. |
| Alexander, James, M.D., M.Ch., B.A. Dub. | Bishop's Place, Paignton, Devonshire. |
| *Alford, George Ernest ... ... ... ... ... | 5 Royal Crescent, Weston-super-Mare. |
| Alford, Henry J., M.D. Lond. ... ... ... | 2 Marlborough Terrace, Taunton. |
| Andrews, Onslow, M.D. St. And.... ... ... | 159 White Ladies Road, Bristol. |
| Anstie, Thomas B. ... ... ... ... ... ... | 21 Northgate Street, Devizes. |
| *Atchley, George F., M.B. Lond.... ... ... | Tyndall's Park, Bristol. |
| *Aveline, H. T. S.... ... ... ... ... ... ... | Bailbrook House, Bath. |
| | |
| Baker, A. de W. ... ... ... ... ... ... ... | 2 Lawn Terrace, Dawlish. |
| *Barclay, W. M. ... ... ... ... ... ... ... | Queen's Road, Clifton. |
| Baretti, T. G. L'Enardi ... ... ... ... ... | 49 Royal York Crescent, Clifton. |
| *Baron, Barclay J., M.B., C.M. Ed. ... ... | 16 White Ladies Road, Clifton. |
| Basset, Walter ... ... ... ... ... ... ... | 24 Stow Hill, Newport, Mon. |
| Batten, Rayner W., M.D. Lond. ... ... ... | 1 Brunswick Square, Gloucester. |
| Batten, W. Smith ... ... ... ... ... ... | Curzon House, Calne. |
| Beale, Lionel S., M.B. Lond., F.R.S. ... | 61 Grosvenor Street, London, W. |
| Beatson, W. B., M.D. St. And. ... ... ... | 11 Cavendish Place, Bath. |
| *Beddoe, John, M.D. Ed., B.A. Lond., F.R.S. | The Manor House, Clifton. |
| Benham, Harry A., M.D., C.M. Aberd. ... | Asylum, Stapleton, Gloucestershire. |
| Bennett, W. S. ... ... ... ... ... ... ... | Escot, Penzance. |
| Berry, F. C., M.D., B.Ch., B.A. Dub. ... ... | Lyn Cottage, Lynton. |
| Bisdée, Alfred J.... ... ... ... ... ... ... | Weston-super-Mare. |
| *Blacker, A. E. ... ... ... ... ... ... ... | Richmond Hill Avenue, Clifton. |

LIST OF SUBSCRIBERS.

| | |
|---|---|
| BLACKER, E. ... ... ... ... ... ... ... ... | Midsomer Norton. |
| *BLAGG, ARTHUR F. ... ... ... ... ... ... | 6 West Mall, Clifton. |
| *BOARD, EDMUND C. ... ... ... ... ... ... | 7 Caledonia Place, Clifton. |
| BOWER, ERNEST D. ... ... ... ... ... ... | 1 Norfolk Terrace, Gloucester. |
| BOYS, ARTHUR HENRY ... ... ... ... ... ... | Chequer Street, St. Alban's. |
| BRAMWELL, J. W., M.D. Ed. ... ... ... ... | 6 Bays Hill Villas, Cheltenham. |
| BRIGHT, J. A. ... ... ... ... ... ... ... ... | Glastonbury. |
| BROOM, JOHN, M.D., Ch.D., Obst.D. Brussels | St. Paul's Road, Clifton. |
| BROWN, G. ARTHUR ... ... ... ... ... ... | Tredegar. |
| BROWN, WILLIAM, M.B., C.M. Glasg. ... ... | Fishponds, Gloucestershire. |
| BROWNE, A. E. NEWBURY ... ... ... ... ... | Burbage, Wiltshire. |
| BROWNE, G. HENRY ... ... ... ... ... ... | The Hermitage, Brynmawr, Breconshire. |
| BROWNE, J. WALTON, M.D., B.A. Qu. Univ. I. | 10 College Square North, Belfast. |
| BROWNE, LENNOX ... ... ... ... ... ... ... | 36 Weymouth Street, London, W. |
| *BURDER, GEORGE F., M.D. Aberd. ... ... ... | 63 Oakfield Road, Clifton. |
| BURGES, R. E., M.D., M.Ch., B.A. Qu. Univ. I. | Kettering. |
| BURROUGHS, E. F. H. ... ... ... ... ... ... | 64 Bristol Road, Weston-super-Mare. |
| *BUSH, J. PAUL ... ... ... ... ... ... ... ... | 13 Lansdown Place, Clifton. |
| *CALDER, F. ... ... ... ... ... ... ... ... | 14 Belle Vue, Cotham, Bristol. |
| *CAMERON, JOHN, M.B., C.M. Glasg. ... ... | 57 Cotham Brow, Bristol. |
| CANN, FRANCIS M. ... ... ... ... ... ... ... | Sefton House, Dawlish. |
| CAREY, J., M.D. Lond. ... ... ... ... ... ... | 2 Hamdon Villas, Taunton. |
| CARLYON, THOMAS B. ... ... ... ... ... ... | Redruth. |
| *CARR, ALEXANDER ... ... ... ... ... ... ... | Wells Road, Bristol. |
| CARTER, R. W., M.D. Calcutta ... ... ... ... | 1 Bank Buildings, Weymouth. |
| *CARTER, W. F.... ... ... ... ... ... ... ... | 48 Coronation Road, Bristol. |
| CAVE, EDWARD J., M.D. Lond. ... ... ... ... | Crewkerne. |
| CLARK, THOMAS ... ... ... ... ... ... ... | Blair, Minehead, Somersetshire. |
| CLARKE, ARTHUR, B.A. Lond. ... ... ... ... | Street, Somersetshire. |
| CLARKE, FRED. H. ... ... ... ... ... ... ... | Holly Lodge, Chillington, Kingsbridge. |
| *CLARKE, JOHN MICHELL, M.B., M.A. Cantab. | 2 York Buildings, Clifton. |
| CLAY, CHALLONER ... ... ... ... ... ... ... | Manor House, Fovant. |
| CLAY, R. HOGARTH, M.D. Ed. ... ... ... ... | 4 Windsor Villas, Plymouth. |
| COATES, HARCOURT ... ... ... ... ... ... | 17 Endless Street, Salisbury. |
| COATHUPE, EDWIN W. ... ... ... ... ... ... | 7 Clifton Park, Clifton. |
| COCK, FREDERICK, M.D. Ed. ... ... ... ... | 1 Porchester Houses, London, W. |
| *COE, R. W. ... ... ... ... ... ... ... ... | 7 Pembroke Road, Clifton. |
| COKER, T. V. ... ... ... ... ... ... ... ... | 2 Chesterfield Place, Clifton. |
| COLE, THOMAS, M.D. Lond. ... ... ... ... | 18 Circus, Bath. |
| *COLLINS, H. W. ... ... ... ... ... ... ... | Wrington, Somersetshire. |
| COLLYNS, ROBERT J. ... ... ... ... ... ... | Dulverton, Somersetshire. |
| COLMAN, THOMAS J., M.D. Ed.... ... ... ... | Elmgrove Road, Bristol. |
| COLMER, PTOLEMY S. H., M.D. Dur. ... ... | South Street House, Yeovil. |
| COOKE, A. S. ... ... ... ... ... ... ... ... | Badbrook House, Stroud. |
| COOPER, HERBERT ... ... ... ... ... ... ... | Hampstead, London, N.W. |
| *CORBOULD, GEORGE G. ... ... ... ... ... | 22 Berkeley Square, Bristol. |
| *COTTON, WILLIAM, M.B., C.M. Ed. ... ... | 227 Gloucester Road, Bristol. |
| COWAN, F. S. ... ... ... ... ... ... ... ... | 27 Queen Square, Bath. |
| COX, WILLIAM I. ... ... ... ... ... ... ... | Hawkesbury-Upton. |
| CRESPI, ALFRED J. H. ... ... ... ... ... | Poole Road, Wimborne. |
| *CROSS, F. RICHARDSON, M.B. Lond. ... ... | Richmond Park Road, Clifton. |
| *CROSSMAN, EDWARD, M.D. Dur. ... ... ... | Hambrook, Gloucestershire. |
| CURRIE, ANDREW S., M.D. Ed. ... ... ... | The Moorlands, Lydney. |

### LIST OF SUBSCRIBERS.

| | |
|---|---|
| *Dacre, John | 2 Leicester Place, Clifton. |
| *Daly, George H., M.D. St. And. | Burley House, Chippenham. |
| Daniel, T. P. | Beaminster, Dorsetshire. |
| *Davey, James G., M.D. St. And. | Mistledene, Surbiton. |
| *Davies, D. S., M.B. Lond. | 60 Oakfield Road, Clifton. |
| Davies, E. Cluneglas | Pontfaen, Lampeter, Cardiganshire. |
| Davies, John | 38 Gay Street, Bath. |
| Davies, John W. | Ebbw Vale, Monmouthshire. |
| Davis, Robert | 14 St. James's Square, Bath. |
| Davis, Theodore, M.D. Lond. | Beachcroft, Clevedon. |
| Day, Percy Howard | Stalmine Lodge, Poulton-le-Fylde. |
| Deas, P. Maury, M.B., M.S. Lond. | Wonford House, Exeter. |
| Dening, Edwin | Stow-on-the-Wold. |
| Devis, Harry F. | Wells Road, Bristol. |
| Devonald, Alfred | Rhoscelyn House, Cowbridge. |
| *Dew, Henry R. | 25 Berkeley Square, Bristol. |
| *Dobson, Nelson C. | 27 Victoria Square, Clifton. |
| *Dowson, W., M.B., B.C., M.A. Cantab. | Great George Street, Bristol. |
| Doyne, Robert W. | Southbourne, Oxford. |
| Duffett, E. D. | Denbigh House, Longton, Staffordshire. |
| Dunbar, Eliza L. Walker, M.D. Zürich | 9 Oakfield Road, Clifton. |
| *Duncan, William | 17 Redland Grove, Bristol. |
| | |
| Eadon, W. F. Bailey | Hambrook, Gloucestershire. |
| *Eager, Reginald, M.D. Lond. | Northwoods, Winterbourne. |
| Eddowes, Charles | Maddington, Devizes. |
| Edgeworth, T. F. | 1 Stokes Croft, Bristol. |
| Edwardes, Thomas | Llansaintffraid, Montgomeryshire. |
| Elder, William | 3 Trelawney Rood, Bristol. |
| *Elliott, Christopher, M.D., B.A. Dub. | 88 Pembroke Road, Clifton. |
| Ellis, Henry V., M.B., C.M. Aberd. | Ty Bryn, Reynoldstone. |
| Ellis, W. Mc. D. | 7 Alexander Buildings, Bath. |
| Embleton, Dennis C. | St. Michael's Road, Bournemouth. |
| *Etches, W. R. | 13 Dowry Square, Clifton. |
| Evans, J. Fenton, M.B., C.M. Ed. | 6 Whitehall Yard, London, S.W. |
| Evans, W. G. | Beckington. |
| *Ewens, John | 14 White Ladies Road, Clifton. |
| | |
| Fairbanks, W., M.D., C.M. Ed. | Wells, Somersetshire. |
| *Fendick, R. George | St. Paul's Road, Clifton. |
| Fernie, James | Stratton St. Margaret. |
| Finch, Thomas, M.D. St. And. | Westville, St. Mary Church. |
| Fisher, Fred. B. | West Walk, Dorchester. |
| Fletcher, Henry Studd | Lydbrook. |
| Ford, Joseph | Wedmore. |
| Forty, D. H. | Wotton-under-Edge. |
| Fox, Arthur E. W., M.B., C.M. Ed. | 16 Gay Street, Bath. |
| *Fox, Bonville B., M.D., M.A. Oxon. | Brislington. |
| Fox, Charles Henry, M.D. St. And. | The Beeches, Brislington. |
| *Fox, E. Long, M.D. Oxon. | Church House, Clifton. |
| Fraser, D. A., M.D. Brussels | Pomeroy House, Totnes. |
| Fraser, Græme B. | Belvedere, Weston-super-Mare. |

| | |
|---|---|
| Freeman, Henry W. | 24 Circus, Bath. |
| Fry, John B. | Ashley Lodge, Esher. |
| Furse, Edwin | South Molton. |
| Fyffe, W. Johnstone, M.D., B.A. Dub. | 2 Rodney Place, Clifton. |
| | |
| Gamble, C. H. | Barnstaple. |
| Gardiner, George | 1 Redcliff Hill, Bristol. |
| *Gibbs, Alfred N. Godby | 27 Chandos Road, Bristol. |
| Gill, John, M.D. Brussels | 31 Apsley Road, Clifton. |
| *Gloag, G. A. | 7 College Green, Bristol. |
| Goodden, Wyndham C. | 86 Stapleton Road, Bristol. |
| Gooding, J. Callender, M.D. Ed. | Berkeley Street, Cheltenham. |
| Goodridge, Henry F. A., M.D. Lond. | 10 Brock Street, Bath. |
| Goss, T. Biddulph | 1 Circus, Bath. |
| Gould, A. Pearce, M.B., M.S. Lond. | 16 Queen Anne Street, London, W. |
| Grace, Alfred | Chipping Sodbury. |
| Grace, Edward Mills | Thornbury, Gloucestershire. |
| Grace, Henry | Kingswood, Bristol. |
| Grace, W. G. | 57 Stapleton Road, Bristol. |
| Gray, F. A. | Ottery St. Mary. |
| Green, F. K. | 3 Gay Street, Bath. |
| Gregory, George S. | Southampton Row, London. |
| *Griffiths, L. M. | 9 Gordon Road, Clifton. |
| Grigor, Macadam | Alexandra Avenue, London, S.W. |
| Grote, G. W., M.B. Toronto | 24 St. Andrew's Crescent, Cardiff. |
| Guinness, T. A. | Bampton, Devonshire. |
| | |
| *Hailes, Clements D. G., M.D., C.M. Ed. | 11 King's Parade, Clifton. |
| *Hale, Edmund T. | The Grange, Chew Magna. |
| Hardwick, A., M.B. Dur. | New Quay, Cornwall. |
| Harper, Joseph | Bear Street, Barnstaple. |
| *Harrison, A. J., M.B. Lond. | Guthrie Road, Clifton. |
| *Harsant, W. H. | 16 Pembroke Road, Clifton. |
| Hawkins, Cæsar F. | 7 Berkeley Square, Bristol. |
| Haydon, Edgar, M.B., C.M. Glasg. | The Laurels, Newton Abbot. |
| Hayman, A. G. | 1 Pembroke Road, Clifton. |
| Heane, W. C. | Cinderford. |
| Hearder, G. J., M.D. St. And. | Joint Counties Asylum, Carmarthen. |
| *Heaven, John C. | 2 Queen Square, Bristol. |
| *Herapath, C. K. C. | 11 Brunswick Square, Bristol. |
| *Hetling, H. E. | Stapleton Road, Bristol. |
| Hett, Geoffrey, M.D. Ed. | 100 Inverness Terrace, London, W. |
| Hichens, James Stacey | 17 Green Lane, Redruth. |
| *Hill, Hedley | General Hospital, Bristol. |
| Hills, Frederic F. | 199 Cheltenham Road, Bristol. |
| Hinton, Joseph | The Chestnuts, Warminster. |
| Hoblyn, Francis Parker | 10 Bladud Buildings, Bath. |
| Hopkins, H. Culliford | 4 Gay Street, Bath. |
| Hospital, Bristol General (*Sec.*, W. Thwaites). | |
| Howells, William, M.B., C.M. Glasg. | Church House, Talgarth, Breconshire. |
| Howse, William | 8 London Street, Swindon, Wiltshire. |
| Howsin, E. Arthur, M.D. St. And. | Stroud. |

### LIST OF SUBSCRIBERS.

| | |
|---|---|
| HUDSON, H. R. ... ... ... ... ... ... ... | 182 Commercial Road, Newport, Mon. |
| HUGGARD, W. R., M.D., M.Ch., M.A. Qu. Univ. I. | Davos Platz, Switzerland. |
| HUNT, A. D. ... ... ... ... ... ... ... ... | Millbrook House, Chagford. |
| HYATT, BROWNLOW N. ... ... ... ... ... ... | Park View, Shepton Mallet. |
| | |
| *IMLAY, G. A., M.B., C.M. Aberd. ... ... ... | 4 Apsley Villas, Bristol. |
| INFIRMARY, BRISTOL ROYAL (*Librarian*, WALTER J. HILL). | |
| INSTITUTION, LIVERPOOL MEDICAL (*Hon. Treas.*, Dr. JAMES BARR). | |
| | |
| JACKSON, J. B., M.D., M. Ch. Roy. Univ. I. ... | 56 Lemon Street, Truro. |
| JAMES, J. BOWEN ... ... ... ... ... ... ... | Woodlands, Aberaman, Aberdare. |
| JESSOP, CHARLES MOORE ... ... ... ... ... | 98 Sutherland Avenue, London, W. |
| JONES, E. R. ... ... ... ... ... ... ... ... ... | Cilgynlle-fawr, New Quay, Cardiganshire. |
| JONES, EVAN ... ... ... ... ... ... ... ... | Ty-Mawr, Aberdare. |
| JONES, J. ARNALT ... ... ... ... ... ... ... | Aberavon. |
| JONES, W. R., M.D., C.M. Glasg. ... ... ... | Senny Bridge, Breconshire. |
| | |
| KEMPE, ARTHUR W. ... ... ... ... ... ... | 9 Southernhay, Exeter. |
| KENDALL, BERNARD ... ... ... ... ... ... | Elm Grove, Tenby. |
| KERR, ELIAS W., M.B., M. Ch., B.A. Dub. ... | Cerne Abbas. |
| KING, LOUIS J. ... ... ... ... ... ... ... ... | 10 Russel Street, Bath. |
| KINNEIR, R. ... ... ... ... ... ... ... ... ... | The Tower House, Malmesbury. |
| KIRKMAN, J. MILLER ... ... ... ... ... ... | Wootton Bassett. |
| | |
| LAING, W. A. GORDON, M.D., C.M. Glasg. ... | Boutport Street, Barnstaple. |
| *LANSDOWN, F. POOLE ... ... ... ... ... ... | 19 White Ladies Road, Clifton. |
| *LAWRENCE, A. E. AUST, M.D., C.M. Aberd. . | 19 Richmond Hill, Clifton. |
| *LAWRENCE, HENRY ... ... ... ... ... ... | 9 Park Row, Bristol. |
| LEACH, J. COMYNS, M.D. Dur., B.Sc. Lond. . | Sturminster Newton. |
| LEAMON, MICHAEL T. ... ... ... ... ... ... | 1 Bedford Villas, Tavistock. |
| *LEES, E. LEONARD, M.B., C.M. Ed. ... ... | Redland Road, Bristol. |
| LEWELLYN, JOHN ... ... ... ... ... ... ... | Caerphilly. |
| LIBRARY, ARMY MEDICAL STAFF ... ... ... | Royal Victoria Hospital, Southampton. |
| LIBRARY, BRISTOL MUSEUM AND ... ... ... | Queen's Road, Bristol. |
| LIBRARY, CHELTENHAM ... ... ... ... ... | 5 Royal Crescent, Cheltenham. |
| *LIDDERDALE, JAMES ... ... ... ... ... ... | Portishead. |
| LIDDON, WILLIAM, M.B. Lond. ... ... ... ... | Church Square, Taunton. |
| LILLEY, G. HERBERT, M.D. Brussels ... ... | H.M. Convict Prison, Portland. |
| LISTER, SIR JOSEPH, Bart., F.R.S. ... ... ... | 12 Park Crescent, London, N.W. |
| LLOYD, WALTER E. ... ... ... ... ... ... | 60 York Road, Bristol. |
| LODGE, JOHN ... ... ... ... ... ... ... ... | Keynsham, Somersetshire. |
| *LOGAN, FREDERIC T. B. ... ... ... ... ... | Dean Lane, Bristol. |
| LOWE, T. PAGAN ... ... ... ... ... ... ... | 2 Belmont Street, Bath. |
| LUCKHAM, L. S. ... ... ... ... ... ... ... | General Infirmary, Salisbury. |
| *LUFFMAN, W. C. ... ... ... ... ... ... ... | 8 Cotham New Road, Bristol. |
| *LYONS, A. DE COURCY, M.B., C.M. Aberd. ... | Henley Lodge, Yatton, Somersetshire. |
| | |
| MACDONALD, P. W., M.D., C.M. Aberd. ... | County Asylum, Dorchester. |
| MACLEAN, J. CAMPBELL, M.B., C.M. Ed. ... | Swindon House, Swindon, Wiltshire. |

| | |
|---|---|
| *Maddison, W. T., M.D. Lond. ... ... ... ... | 15 Aberdeen Terrace, Bristol. |
| Manning, H. J., B.A. Lond. ... ... ... ... | Laverstock House, Salisbury. |
| Marley, Henry F. ... ... ... ... ... ... | The Nook, Padstow, Cornwall. |
| Marsh, Charles J. ... ... ... ... ... ... | Yeovil. |
| Marsh, O. E. Bulwer ... ... ... ... ... | Ventnor House, Newport, Mon. |
| Marshall, Arthur L. ... ... ... ... ... | Royal United Hospital, Bath. |
| *Marshall, Henry, M.D. Ed., F.R.S.E. ... | 28 Caledonia Place, Clifton. |
| *Martin, E. F., M.B. Ed. ... ... ... ... ... | Victoria House, Weston-super-Mare. |
| Martin, Theodore ... ... ... ... ... ... | Temple Cloud. |
| Mason, S. B. ... ... ... ... ... ... ... ... | 12 Park Terrace, Pontypool. |
| Mason, William ... ... ... ... ... ... ... | Sedgemoor Cottage, St. Austell. |
| *Masters, W. Hooper, M.D. St. And. ... ... | 6 Kensington Place, Clifton. |
| *Matthews, Charles E. ... ... ... ... ... | 31 Pembroke Road, Clifton. |
| *Mayor, T. O. ... ... ... ... ... ... ... ... | 40 Apsley Road, Clifton. |
| McNicoll, John ... ... ... ... ... ... ... | High Street, Poole. |
| Meredith, John, M.D. Ed. ... ... ... ... | Wellington, Somersetshire. |
| Metford, J. Seymour ... ... ... ... ... | 34 West Mall, Clifton. |
| Michell, George A. ... ... ... ... ... ... | Redruth. |
| Milne, John, M.B., C.M. Aberd. ... ... ... | 97 Clarence Road, Bristol. |
| Milward, James, M.D. Brussels ... ... ... | 54 Charles Street, Cardiff. |
| Monckton, William ... ... ... ... ... ... | Portishead. |
| Morgan, Frederick ... ... ... ... ... ... | Uffculme. |
| Morgan, John ... ... ... ... ... ... ... ... | Langport, Somersetshire. |
| Morgan, William, M.D. Aberd. ... ... ... | 16 Edwards Terrace, Cardiff. |
| *Morton, C. A.... ... ... ... ... ... ... ... | Zetland Road, Bristol. |
| Moynan, W. Arthur, M.D., M.Ch.Qu. Univ. I. | Wyke House, Isleworth. |
| Munden, Charles ... ... ... ... ... ... ... | Ilminster. |
| Murray, William, M.B., C.M. Ed. ... ... | Staple Hill, Gloucestershire. |
| | |
| Napier, T. W. A., M.B., C.M. Aberd. ... ... | Church Street, Egremont, Cheshire. |
| *Newman, Charles ... ... ... ... ... ... ... | 19 Portland Square, Bristol. |
| *Newnham, W. H. C., M.B., M.A. Cantab. ... | General Hospital, Bristol. |
| Newstead, James ... ... ... ... ... ... ... | 9 York Place, Clifton. |
| Newton, Charles John ... ... ... ... ... | Oriel Lodge, Cheltenham. |
| Nicholson, T. D., M.D., C.M. Ed. ... ... | 2 White Ladies Road, Clifton. |
| Norman, J. H. ... ... ... ... ... ... ... ... | Goodleigh, Winkleigh, Devonshire. |
| Norman, J. W.... ... ... ... ... ... ... ... | Edde Cross House, Ross. |
| *Norton, J. A., M.D., C.M. Aberd. ... ... ... | 8 Redcliff Hill, Bristol. |
| | |
| Ollerhead, T. J. ... ... ... ... ... ... ... | Hillbury, Minehead, Somersetshire. |
| *Ormerod, Henry ... ... ... ... ... ... ... | Westbury-on-Trym. |
| | |
| Padley, George ... ... ... ... ... ... ... | 2 Northampton Terrace, Swansea. |
| Page, G. Shepley ... ... ... ... ... ... ... | 78 Old Market Street, Bristol. |
| Paine, William Henry, M.D. Aberd. ... ... | Stroud. |
| Palmer, F. Craddock, M.D. Brussels ... ... | The Corderries, Chalford. |
| *Parker, George, M.D., M.A. Cantab. ... ... | 14 Pembroke Road, Clifton. |
| Parry, J. H. ... ... ... ... ... ... ... ... | 99 Ashley Road, Bristol. |
| Parsloe, H. H. ... ... ... ... ... ... ... | Hatherleigh Devonshire. |
| *Parson, T. Cooke ... ... ... ... ... ... ... | 21 White Ladies Road Bristol. |

### LIST OF SUBSCRIBERS.

| | |
|---|---|
| PARSONS, FRANCIS J. C. ... ... ... ... ... ... | King Square, Bridgwater. |
| PARSONS, FREDERICK ... ... ... ... ... ... ... | Common Hill, Frome. |
| PATERSON, DONALD ROSE, M.D., C.M. Ed.... | 7 Newport Road, Cardiff. |
| PAULI, G. C. P. ... ... ... ... ... ... ... ... | 240 York Road, Bristol. |
| PAULL, JOSIAH ... ... ... ... ... ... ... ... ... | Camborne, |
| *PENNY, F. ... ... ... ... ... ... ... ... ... ... | 42 Caledonia Place, Clifton. |
| *PENNY, W. J. ... ... ... ... ... ... ... ... ... | 6 Chandos Street, London, W. |
| PENTLAND, YOUNG J. ... ... ... ... ... ... ... | 11 Teviot Place, Edinburgh. |
| PERKINS, J. H., M.D. New York ... ... ... | 14 Victoria Square, Clifton. |
| PESKETT, A. W. C., M.B., B.C., M.A. Cantab. | Burnham, Somersetshire. |
| PHELPS, W. HARFORD G., M.D. Aberd. ... | Beachfield, Weston-super-Mare. |
| PHILLIPS, E. ENGLAND ... ... ... ... ... ... | Broadwater House, Southend. |
| *PICKERING, C. F. ... ... ... ... ... ... ... ... | 12 Berkeley Square, Bristol. |
| PIPPETTE, WALTER... ... ... ... ... ... ... ... | 90 Brooke Road, London, N. |
| PIZEY, G. F. P.... ... ... ... ... ... ... ... ... | Rydal Villa, Clevedon. |
| PLAISTER, W. H. ... ... ... ... ... ... ... ... | High Road, Tottenham. |
| *POUNTNEY, W. E., M.D., C.M. Ed. ... ... | 107 White Ladies Road, Bristol. |
| POWELL, WILLIAM, M.B. Lond. ... ... ... | Hill Garden, Torquay. |
| PRICE, WILLIAM, M.B. Lond. ... ... ... ... | 40 Park Place, Cardiff. |
| *PRICHARD, ARTHUR W.... ... ... ... ... ... | Gordon Road, Clifton. |
| *PRICHARD, AUGUSTIN, M.D. Berlin... ... ... | 4 Chesterfield Place, Clifton. |
| *PRICHARD, J. E., M.B., B.A. Oxon. ... ... | 243 Cheltenham Road, Bristol. |
| PRIDEAUX, T. ENGLEDUE P. ... ... ... ... | Wellington, Somersetshire. |
| *PRING, ARTHUR, B.A. Cantab. ... ... ... ... | 4 Pembroke Road, Clifton. |
| PROWSE, ALBERT P. ... ... ... ... ... ... ... | Yennadon, Horrabridge, Devonshire. |
| *PROWSE, ARTHUR B., M.D. Lond. ... ... ... | 5 Lansdown Place, Clifton. |
| | |
| RANDOLPH, CHARLES ... ... ... ... ... ... ... | St. Michael's, Milverton, Somersetshire. |
| RAWLINGS, JOHN ADAMS ... ... ... ... ... ... | 4 Northampton Terrace, Swansea. |
| REDWOOD, T. HALL, M.D., C.M., M.A. Dur.... | The Lawn, Rhymney, Monmouthshire. |
| RENDALL, JOHN... ... ... ... ... ... ... ... ... | Forestside, Lymington. |
| RIDDELL, ANDREW W. ... ... ... ... ... ... ... | Westbury-on-Trym. |
| ROBATHAN, G. B. ... ... ... ... ... ... ... ... | The Grove, Risca. |
| ROLSTON, G. T. ... ... ... ... ... ... ... ... | 8 Osborne Villas, Stoke, Devonport. |
| *ROSS, R. A. ... ... ... ... ... ... ... ... ... | Lodway Villa, Pill. |
| *ROSSITER, GEORGE F., M.B. Lond. ... ... | Cairo Lodge, Weston-super-Mare. |
| *ROUÉ, W. BARRETT, M.D., M.S. Dur. ... ... | 2 Buckingham Place, Clifton. |
| ROWE, EDMUND L.... ... ... ... ... ... ... ... | County Asylum, Gloucester. |
| *ROXBURGH, ROBERT, M.B. Ed.... ... ... ... | 1 Victoria Buildings, Weston-super-Mare. |
| ROYLE, JOHN FREDERICK S., M.B., C.M. Aberd. | 30 Foregate Street, Worcester. |
| ROYSTON, CHARLES, M.D. St. And. ... ... | 1 St. Stephen's Crescent, London, W. |
| *RUDGE, C. KING ... ... ... ... ... ... ... ... | 145 White Ladies Road, Bristol. |
| *RUDGE, H. T. ... ... ... ... ... ... ... ... ... | 5 Colston Parade, Bristol. |
| RUNDLE, EDMUND ... ... ... ... ... ... ... ... | Royal Cornwall Infirmary, Truro. |
| RUSSELL, M. W. H. ... ... ... ... ... ... ... | 6 Whitehall Yard, London, S.W. |
| | |
| SCOTT, JAMES, M.B., C.M. Ed.... ... ... ... | 35 Wolverhampton Road, Stafford. |
| SCOTT, RICHARD J. H. ... ... ... ... ... ... | 13 Bladud Buildings, Bath. |
| SCOTT, W. G., M.B., C.M. Ed. ... ... ... | Newton Abbot. |
| SEARLE, GEORGE C. ... ... ... ... ... ... ... | Brixham, Devonshire. |

### LIST OF SUBSCRIBERS.

| | |
|---|---|
| Searle, Richard Burford ... ... ... ... | St. Just. |
| *Semple, H. F. ... ... ... ... ... ... ... ... | Swellendam, South Africa. |
| Seward, W. J., M.B. Lond. ... ... ... ... | Asylum, Colney Hatch, London, N. |
| Sewart, J. H. ... ... ... ... ... ... ... ... | Lostwithiel. |
| Shapter, Lewis, M.D., B.A. Cantab. ... ... | The Barnfield, Exeter. |
| *Shaw, J. E., M.B., C.M. Ed. ... ... ... ... | 11 Lansdown Place, Clifton. |
| Sheen, A., M.D. St. And. ... ... ... ... ... | 23 Newport Road, Cardiff. |
| *Sheppard, E. J. ... ... ... ... ... ... ... | 16 Park Place, Clifton. |
| *Shorland, Walter E. ... ... ... ... ... | 189 Cheltenham Road, Bristol. |
| Shortridge, Thomas Wood, M.D. Brussels | Honiton. |
| Sincock, J. Bain ... ... ... ... ... ... ... | Friarn Street, Bridgwater. |
| Skeate, Edwin ... ... ... ... ... ... ... | 16 Paragon, Bath. |
| *Skelton, Henry, M.D. St. And. ... ... ... | Downend, Gloucestershire |
| *Skerritt, E. Markham, M.D., B.S., B.A. Lond. | Richmond Hill, Clifton. |
| *Skinner, Stephen, M.B., C.M. Aberd. ... | Ferndale, Clevedon. |
| Smith, D. Boyes, M.D. Ed. ... ... ... ... | Eastcliff, Woolstone, Southampton. |
| *Smith, G. Munro ... ... ... ... ... ... ... | 70 Pembroke Road, Clifton. |
| *Smith, J. Greig, M.B., C.M., M.A. Ab., F.R.S.E. | 16 Victoria Square, Clifton. |
| Smith, J. Sidney, M.D. St. And. ... ... ... | Tiverton. |
| *Smith, R. Shingleton, M.D., B.Sc. Lond. | Clifton Park, Clifton. |
| Smith, Samuel ... ... ... ... ... ... ... | 7 Kingsdown Parade, Bristol. |
| Society, Cardiff Medical (*Hon. Sec.*, C. E. | Hardyman). |
| Society, Royal Medical and Chirurgical | 53 Berners Street, London, W. |
| Somer, James ... ... ... ... ... ... ... ... | Broadclyst. |
| Spencer, H. A. ... ... ... ... ... ... ... | Royal United Hospital, Bath. |
| *Spencer, W. H., M.D., M.A. Cantab. ... ... | 11 Warrior Square, St. Leonard's-on-Sea. |
| Spender, John K., M.D. Lond. ... ... ... | 17 Circus, Bath. |
| Stansfeld, G. M. ... ... ... ... ... ... ... | 19 Victoria Square, Clifton. |
| Statham, R. Whiteside ... ... ... ... ... | Cheddar, Somersetshire |
| Steel, Arthur R.... ... ... ... ... ... ... | Fern Villa, Belle Vue, Wakefield. |
| Steel, S. H., M.B. Lond. ... ... ... ... ... | Abergavenny. |
| *Steele, Charles, M.D. Dur. ... ... ... ... | 17 Richmond Hill, Clifton. |
| Steele-Perkins, E. B. ... ... ... ... ... | 1 Hill's Buildings, St. Sidwell's, Exeter. |
| Stephens, Lockhart E. W. ... ... ... ... | Emsworth. |
| Stewart, James, B.A. Qu. Univ. I. ... ... | Dunmurry, Stoke Bishop. |
| Stockwell, Fred., M.D. Lond. ... ... ... | Bruton, Somersetshire. |
| Stoddart, F. Wallis ... ... ... ... ... ... | 1 Park Street, Bristol. |
| Storry, Frederick W. ... ... ... ... ... | Lansdown, Stroud. |
| *Swain, James, M.B., B.S. Lond. ... ... ... | Royal Infirmary, Bristol. |
| *Swayne, J. G., M.D. Lond. ... ... ... ... | 74 Pembroke Road, Clifton. |
| *Swayne, S. H.... ... ... ... ... ... ... ... | 129 Pembroke Road, Clifton. |
| Sydenham, George F. ... ... ... ... ... | Brushford, Exbridge. |

| | |
|---|---|
| Tait, Lawson ... ... ... ... ... ... ... ... | 7 The Crescent, Birmingham |
| *Taylor, James ... ... ... ... ... ... ... ... | 36 Alma Road, Clifton. |
| Taylor, Thomas Henry ... ... ... ... ... | Thornbury, Gloucestershire. |
| Thomas, A. Garrod, M.D., C.M. Ed. ... ... | Clytha Park, Newport, Mon. |
| Thomas, G. Danford, M.D. Brussels ... ... | Park Lodge, Paddington, London, W. |
| Thomas, Jabez ... ... ... ... ... ... ... | Ty Cerrig, Swansea. |
| Thomas, R. W. ... ... ... ... ... ... ... | Freemantle Square, Bristol. |
| Thompson, George, M.D. Dur. ... ... ... | Asylum, Stapleton, Gloucestershire. |
| Thomson, G. S., M.D. Ed. ... ... ... ... | 8 College Road, Clifton. |
| *Tivy, W. J. ... ... ... ... ... ... ... ... | 8 Lansdown Place, Clifton. |

### LIST OF SUBSCRIBERS.

| | |
|---|---|
| Todd, W. J. ... ... ... ... ... ... ... ... ... | North Petherton. |
| *Tomkins, Harding H.... ... ... ... ... ... ... | 3 White Ladies Road, Clifton. |
| Torbock, W. H., M.D. Pennsylvania ... ... | Polruan, Cornwall. |
| Tosswill, Louis H., M.B., B.A. Cantab. ... | 28 West Southernhay, Exeter. |
| Trotter, Leslie B., M.D., C.M. Ed. ... ... | Coleford, Gloucestershire. |
| Twining, A. H. ... ... ... ... ... ... ... ... | The Knoll, Salcombe. |
| Tyley, R. P., M.D. St. And. ... ... ... ... | Wedmore. |
| | |
| Vachell, C. T., M.D. Lond. ... ... ... ... | 38 Charles Street, Cardiff. |
| Vachell, Herbert R., M.D., C.M. Aberd.... | 15 Newport Road, Cardiff. |
| Vernon, Edward ... ... ... ... ... ... ... ... | Kingston, Yeovil. |
| | |
| Wade, A. Law, M.D., B.A. Dub. ... ... ... | County Asylum, Wells, Somersetshire. |
| *Waldo, Henry, M.D., C.M. Aberd. ... ... | 19 Pembroke Road, Clifton. |
| Wallace, Rev. C. Hill, M.A. Oxon. ... ... | 3 Harley Place, Clifton. |
| Walter, William Henry, M.D. Brussels ... | Knap Villa, South Petherton. |
| Ward, J. L. W. ... ... ... ... ... ... ... ... | Victoria Street, Merthyr Tydvil. |
| *Wathen, J. Hancocke ... ... ... ... ... ... | 16 York Place, Clifton. |
| Watson, J. Adam ... ... ... ... ... ... ... ... | 39 Dennington Park, London, N.W. |
| Watters, George T. B., M.D., C.M. Ed. ... | Stonehouse, Gloucestershire. |
| Waugh, Alexander ... ... ... ... ... ... ... | Midsomer Norton. |
| *Weatherly, Lionel A., M.D., C.M. Aberd. | Bailbrook House, Bath. |
| Webber, William W.... ... ... ... ... ... ... | Crewkerne. |
| *Webster, Thomas ... ... ... ... ... ... ... | Redland Hill, Bristol. |
| *Wedmore, C. Ernest, M.B. Cantab. ... ... | 11 Richmond Hill, Clifton. |
| *Welsh, F. F. ... ... ... ... ... ... ... ... ... | Rockleaze Point, Stoke Bishop. |
| Whishaw, Reginald R., M.B., B.C., B.A. Cantab. | St. Paul's Road, Clifton. |
| Whiteley, George W. ... ... ... ... ... ... | Hamilton House, Downton, Wiltshire. |
| Whitfeld, W. Clarke ... ... ... ... ... ... | 5 St. Ethelbert Street, Hereford. |
| Whyte, Andrew, M.D., C.M. Aberd. ... ... | Honddû House, Brecon. |
| Wickham, W. ... ... ... ... ... ... ... ... ... | Hill House, Tetbury. |
| Wicksteed, Francis W. S. ... ... ... ... | The Villa Rosa, Weston-super-Mare. |
| Wigmore, J., M.D. Qu. Univ. I.... ... ... ... | Twerton-on-Avon. |
| *Wilding, James, M.D. Dur. ... ... ... ... | Sydenham Road, Bristol. |
| Willcox, R. Lewis ... ... ... ... ... ... ... | Warminster. |
| Willett, George G. D. ... ... ... ... ... | Keynsham. |
| Williams, Charles ... ... ... ... ... ... ... | Port Isaac. |
| Williams, D. J. ... ... ... ... ... ... ... ... | Greenfield House, Llanelly. |
| Williams, H. Egerton ... ... ... ... ... ... | The Mount, Newport, Mon. |
| *Williams, P. Watson, M.B. Lond. ... ... | 24 Pembroke Road, Clifton. |
| Williams, Peter ... ... ... ... ... ... ... ... | Ferryside, Carmarthenshire. |
| Williams, William Henry, M.D. Lond. ... | Sherborne. |
| Wilson, Claude, M.D., C.M. Ed. ... ... ... | Church Road, Tunbridge Wells. |
| *Windsor-Aubrey, H. W. ... ... ... ... ... | 3 Portland Square, Bristol. |
| Winterbotham, L. ... ... ... ... ... ... ... | Bays Hill, Cheltenham. |
| Wood, E. Stanley ... ... ... ... ... ... ... | Clarence House, Pontypool. |
| Woodd, H. T. ... ... ... ... ... ... ... ... ... | Kelly Villa, Calstock. |
| | |
| Yelf, Leonard K., M.D. St. And. ... ... ... | Moreton-in-Marsh. |

### Exchange-List.

### AFRICA.

### CAPE COLONY.

*Weekly.*

*The South African Medical Journal.* East London: W. Darley-Hartley.

### AMERICA.

### ARGENTINE REPUBLIC.

*Monthly.*

*Anales del Circulo Médico Argentino.* Buenos Ayres: M. Biedma.

### CANADA.

*Monthly.*

*The Montreal Medical Journal.* Montreal: Gazette Printing Company.

### PERU.

*Monthly.*

*La Crónica Médica.* Lima: Carlos Prince.

### UNITED STATES.

*Weekly.*

*The Cincinnati Lancet-Clinic.* Cincinnati: 281 West 7th Street.

*The Journal of the American Medical Association.* Chicago: 65 Randolph Street.

*The Medical and Surgical Reporter.* Philadelphia: 115 South Seventh Street.

*The Medical Record.* New York: William Wood & Co.

*The New York Medical Journal.* New York: D. Appleton & Co.

*Fortnightly.*

*The American Practitioner & News.* Louisville: John P. Morton and Company.

*Twice a month.*

*The Medical Age.* Detroit: George S. Davis.

*Monthly.*

*Index Medicus.* Detroit: George S. Davis.

*Journal of Cutaneous and Genito-Urinary Diseases.* New York: D. Appleton & Co.

*New Orleans Medical and Surgical Journal.* New Orleans: 33 Carondelet Street.

*Pacific Medical and Surgical Journal and Western Lancet.* San Francisco: Bonestell & Co.

*The Abstract and Index.* Weston: H. H. Howe.

*The American Journal of Ophthalmology.* St. Louis: J. H. Chambers & Co.

*Monthly.*

*The American Journal of the Medical Sciences.* Philadelphia: Lea Brothers & Co.

*The Medical Analectic.* New York: G. P. Putnam's Sons.

*The Obstetric Gazette.* Cincinnati: 281 West 7th Street.

*The Saint Louis Medical and Surgical Journal.* Saint Louis: 2622 Washington Avenue.

*The Therapeutic Gazette.* Detroit: George S. Davis.

*Yearly.*

*Annual of the Universal Medical Sciences.* Philadelphia: F. A. Davis.

*Transactions of the American Surgical Association.* Philadelphia: P. Blakiston, Son & Co.

*Occasionally.*

*Transactions of the New York Academy of Medicine.* New York: 12 West 31st Street.

### ASIA.

### INDIA.

*Monthly.*

*The Indian Medical Journal.* Calcutta: W. Newman & Co., Ld.

### JAPAN.

*Monthly.*

*The Sei-I-Kwai Medical Journal.* Tokio: Z. P. Maruya & Co.

### AUSTRALASIA.

### AUSTRALIA.

*Monthly.*

*The Australasian Medical Gazette.* Sydney: L. Bruck.

*The Australian Medical Journal.* Melbourne: Stillwell & Co.

### EUROPE.

### BELGIUM.

*Monthly.*

*Bulletin de l'Académie royale de médecine de Belgique.* Brussels: F. Hayez.

### BRITISH ISLES.

*Weekly.*

*British Medical Journal.* London: 429 Strand.

*The Hospital.* London: 38 Old Jewry.

*The Lancet.* London: 423 Strand.

*Monthly.*

*Annals of Surgery.* London: Baillière, Tindall & Cox.

*The Birmingham Medical Review.* Birmingham: Cornish Bros.

*The Journal of Laryngology and Rhinology.* London: R. Anderson & Co.

*The London Medical Recorder.* London: W. H. Allen & Co.

*The Medical Chronicle.* Manchester: John Heywood.

*The Practitioner.* London: Macmillan & Co.

*Edinburgh Medical Journal.* Edinburgh: Oliver and Boyd.

*The Glasgow Medical Journal.* Glasgow: Alex. Macdougall.

*The Dublin Journal of Medical Science.* Dublin: Fannin & Co.

*Quarterly.*

*The Asclepiad.* London: Longmans, Green, & Co.

*The British Gynæcological Journal.* London: John Bale & Sons.

*Half-yearly.*

*The Liverpool Medico-Chirurgical Journal.* Liverpool: Medical Institution.

*The Retrospect of Medicine.* London: Simpkin, Marshall, & Co.

*Yearly.*

*The Medical Annual.* Bristol: John Wright & Co.

*The Year-Book of Treatment.* London: Cassell & Company, Limited.

*Year-Book of Scientific and Learned Societies.* London: C. Griffin & Co.

*Transactions of the Academy of Medicine in Ireland.* Dublin: Fannin and Company.

DENMARK.

*Weekly.*

*Hospitals-Tidende.* Copenhagen: Jacob Lund.

FRANCE.

*Three times a week.*

*Gazette des hôpitaux.* Paris: 4 Rue de l'Odéon.

*L'Union médicale.* Paris: 11 Rue de la Grange-Batelière.

*Weekly.*

*Bulletin de l'Académie de médecine.* Paris: G. Masson.

*Le Progrès médical.* Paris: 14 Rue des Carmes.

*Twice a month.*

*Gazette de Gynécologie.* Paris: O. Doin.

*Monthly.*

*Nouvelles Archives d'Obstétrique et de Gynécologie.* Paris: Félix Alcan.

*Revue mensuelle de Laryngologie, d'Otologie et de Rhinologie.* Paris: 8 Place de l'Odéon.

GERMANY.

*Twice a week.*

*Deutsche Medizinal-Zeitung.* Berlin: Eugen Grosser.

*Weekly.*

*Medicinische Bibliographie.* Leipzig: Breitkopf & Härtel.

HOLLAND.

*Weekly.*

*Nederlandsch Tijdschrift voor Geneeskunde.* Amsterdam: F. van Rossen.

ITALY.

*Daily.*

*La Riforma Medica.* Naples: 48 Sette Dolori a Toledo.

*Monthly.*

*Lo Sperimentale.* Florence: 35 Via degli Alfani.

NORWAY.

*Monthly.*

*Norsk Magazin for Lægevidenskaben.* Christiania: Th. Steens.

PORTUGAL.

*Weekly.*

*A Medicina Contemporanea.* Lisbon: José Antonio Rodrigues & Ca.

ROUMANIA.

*Monthly.*

*Spitalul.* Bucharest: Stefan Mihalescu.

RUSSIA.

*Weekly.*

*Vrach.* St. Petersburg: C. Ricker.

SPAIN.

*Weekly.*

*El Siglo Médico.* Madrid: Magdalena, 36, 2º

SWEDEN.

*Quarterly.*

*Nordiskt Medicinskt Arkiv.* Stockholm: P. A. Norstedt & Söner.

SWITZERLAND.

*Monthly.*

*Illustrirte Monatsschrift der ärztlichen Polytechnik und Centralblatt der orthopädischen Chirurgie.* Berne: Schmid, Francke & Co.

### *PUBLICATIONS RECEIVED.*

From The Sei-I-Kwai, Tōkyō:
*The Catalogue of the Tōkyō Medical Library.* 1888.
From Rockwell & Churchill, Boston:
*Periodicals, Transactions, and Reports in the Library of the New York Academy of Medicine.* Part I. United States and Gt. Britain. 1889.
From John Carnrick, New York:
*The Dietetic Gazette.* October, December, 1888.
From 95 William Street, New York:
*The International Journal of Surgery and Antiseptics.* Vol. I., No. 4. October, 1888. Vol. II. Nos. 1, 2. January, February, 1889.
From P. Blakiston, Son & Co., Philadelphia:
*The Polyclinic.* December, 1888—February, 1889.
From Wm. J. Dornan, Philadelphia:
*Contributions to the Anatomy and Physiology of the Thymus Gland.* By A. Jacobi, M.D. 1888.
From Lea Brothers & Co., Philadelphia:
*Poisoning by Chrome-Yellow used as a Cake Dye.* By David Denison Stewart, M.D. 1889. Reprint.
From A. J. Johnston & Co., Sacramento:
*Occidental Medical Times.* Vol. III. No. 1. January, 1889.
From 3860 Pine Street, St. Louis:
*The Alienist and Neurologist.* Vol. X. No. 1. January, 1889.
From Emile Lecrosnier et Babé, Paris:
*Nouveaux Faits confirmant l'efficacité de l'Électrolyse lineaire dans le traitement des Rétrécissements de l'Urèthre.* Par le Dr. J. A. Fort. 1888.
From 5 Avenue de l'Opéra, Paris:
*Annales d'Orthopédie et de Chirurgie pratiques.* Tome III. Nos. 3–5. 1889.
From Eugen Grosser, Berlin:
*Das Höhenklima in meteorologischer, physiologischer und therapeutischer Beziehung.* Von Dr. August Ladendorf. 1889.
From Young J. Pentland, Edinburgh:
*Reports from the Laboratory of the Royal College of Physicians, Edinburgh.* Edited by J. Batty Tuke, M.D., and G. Sims Woodhead, M.D. Vol. I. 1889.
From Christy & Co., London:
*New Commercial Plants and Drugs.* No. 11. By T. Christy. 1889.
From J. & A. Churchill, London:
*Cancer and Cancerous Diseases.* By Sir Spencer Wells, Bart. 1889.
From Harrison and Sons, London:
*Defective Articulation resulting from Cleft Palate.* By William Van Praagh. Reprint. 1888.
From H. K. Lewis, London:
*The British Journal of Dermatology.* Vol. I. Nos. 2–5. December, 1888—March, 1889.
*Masso-Therapeutics.* By William Murrell, M.D. Fourth Ed. 1889.
*The Causation of Disease.* By Harry Campbell, M.D., B.S. 1889.
*Epitome of Surgery.* By Ridley Dale, M.D. 1889.
*Human Physiology.* By Austin Flint, M.D., LL.D. Fourth Ed. 1888.
*Congestive Neurasthenia.* By E. G. Whittle, M.D. 1889.
*Diabetes.* By Emil Schnée, M.D. Translated by R. L. Tafel, A.M., Ph.D. 1889.
*Diseases of Women.* By Alexander J. C. Skene, M.D. 1889.
*The Treatment of Inebriety in the Higher and Educated Classes.* By James Stewart, B.A. 1889.
*The Middlesex Hospital. Reports of the Medical, Surgical, and Pathological Registrars for the year* 1887. 1888.
From Simpkin & Marshall, London:
*A Pamphlet on Vaccination.* By Wm. Woodward, M.D. 1888.
From A. A. Tindall, London:
*The Medical Press and Circular.* January 2, 1889.
From Trübner & Co., London:
*The Therapeutics of Kronenquelle Water.* By Prosser James, M.D. 1889.

Reviews of Books ... 44

Notes on Preparations for the Sick ... 56

Periscope ... 60

Scraps ... 71

*Cases for binding, price 7d. each, may be had of the Publisher,*
J. W. Arrowsmith, *Quay Street, Bristol.*

